# Supplementary material for: Artificial intelligence with temporal features outperforms machine learning in predicting diabetes
Source: PLOS Digit Health. 2023 Oct 25;2(10):e0000354. doi: 10.1371/journal.pdig.0000354 (PMC10599553; doi:10.1371/journal.pdig.0000354)
Supplement: Attachment — Submitted filename: Response Letter - PLOS Digital Health - Signed KK.pdf [file pdig.0000354.s001.pdf]

Institute of Health Policy, Management & Evaluation
UNIVERSITY OF TORONTO

The Editor
PLOS Digital Health

July 8, 2023

Dear Editor,

Thank you for the opportunity to submit our revised manuscript to PLOS Digital Health. Reviewer #2 had a few requests for changes to our submitted manuscript. We have placed the reviewer's comments and our disposition in the table below.

Reviewer Comment	Our response
1. Summarize the recent works in the form of a table.	Done
2. The model's performance is evaluated using accuracy, specificity and sensitivity, precision and f1-score but the formulas for precision and F1-score are not defined in section 2.7. include them accordingly.	Added into section 2.7
3. Compare and explain the significance of using machine learning and deep learning in diabetes prediction.	Already explained in section 4 Discussion.
4. Give a comparison of earlier reported work with current work in the Discussion section.	Done
5. What is the benefit of the designed method? Explain	Added to section 4 Discussion.
6. The Tuning number of layers in machine and deep learning models is not clear, kindly revise it and add more explanations.	Added to section 2.5 A and B. We used the term Epochs instead of tuning layers.
7. Make a statistical test to compare the results against the methods in literature for the data sets presented.	This request is not at all clear. We have provided several important statistical tests, including sensitivity, specificity, precision and F1 scores. These are the standard tests for these types of studies.
8. Improve the English language for enhanced the quality of the paper.	Done –in tracked changes
9. Aggregate articles from the journal " PLOS Digital Health".	This is not an appropriate recommendation.

We trust that these responses are satisfactory and that you can publish the paper soon.

Once again, thank you for the opportunity to submit an article to PLOS Digital Health.

Sincerely,

KKeshavjee

Karim Keshavjee MD, MBA
Assistant Professor, Teaching Stream
Program Director, Health Informatics
Institute for Health Policy, Management and Evaluation
Dalla Lana School of Public Health
University of Toronto
