# Supplementary material for: Artificial intelligence with temporal features outperforms machine learning in predicting diabetes
Source: PLOS Digit Health. 2023 Oct 25;2(10):e0000354. doi: 10.1371/journal.pdig.0000354 (PMC10599553; doi:10.1371/journal.pdig.0000354)
Supplement: Attachment — Submitted filename: Minor reviews 17-8-23.docx [file pdig.0000354.s002.docx]

**REVIEWS (MINOR REVISION)**

**PDIG-D-23-00121R1 for PLOS Digital Health entitled Artificial intelligence with temporal features outperforms machine learning in diabetes prediction .**

Following suggestion need to be addressed:

sensitivity, specificity, precision and F1 scores are not statistical tests but are evaluation measures which are used t*o evaluate the performance or quality of the model, and these metrics are known as performance metrics or evaluation metrics.*

Statistical tests are a way of mathematically determining whether two sets of data are significantly different from each other. There are various statistical tests that can be used, depending on the type of data being analyzed. However, some of the most common statistical tests are chi-squared tests, ANOVA test. t-test, Z-test, chi-square test, binomial test and so on….
